# Flu@home: the Comparative Accuracy of an At-Home Influenza Rapid Diagnostic Test Using a Prepositioned Test Kit, Mobile App, Mail-in Reference Sample, and Symptom-Based Testing Trigger

**DOI:** 10.1128/jcm.02070-21

**Published:** 2022-03-16

**Authors:** Jack Henry Kotnik, Shawna Cooper, Sam Smedinghoff, Piyusha Gade, Kelly Scherer, Mitchell Maier, Jessie Juusola, Ernesto Ramirez, Pejman Naraghi-Arani, Victoria Lyon, Barry Lutz, Matthew Thompson

**Affiliations:** a Department of Bioengineering, University of Washington, Seattle, Washington, USA; b Department of Family Medicine, University of Washington, Seattle, Washington, USA; c Audere, Seattle, Washington, USA; d Evidation Health, Inc., San Mateo, California, USA; e Tunnell Government Services, Inc., Bethesda, Maryland, USA; Mayo Clinic

**Keywords:** RDT, SARS-CoV-2, antigen test, at-home testing, comparative accuracy, diagnostic accuracy, diagnostic performance, influenza, rapid tests, respiratory viruses

## Abstract

At-home testing with rapid diagnostic tests (RDTs) for respiratory viruses could facilitate early diagnosis, guide patient care, and prevent transmission. Such RDTs are best used near the onset of illness when viral load is highest and clinical action will be most impactful, which may be achieved by at-home testing. We evaluated the diagnostic accuracy of the QuickVue Influenza A+B RDT in an at-home setting. A convenience sample of 5,229 individuals who were engaged with an on-line health research platform were prospectively recruited throughout the United States. “Flu@home” test kits containing a QuickVue RDT and reference sample collection and shipping materials were prepositioned with participants at the beginning of the study. Participants responded to daily symptom surveys. If they reported experiencing cough along with aches, fever, chills, and/or sweats, they used their flu@home kit following instructions on a mobile app and indicated what lines they saw on the RDT. Of the 976 participants who met criteria to use their self-collection kit and completed study procedures, 202 (20.7%) were positive for influenza by qPCR. The RDT had a sensitivity of 28% (95% CI = 21 to 36) and specificity of 99% (98 to 99) for influenza A, and 32% (95% CI = 20 to 46) and 99% (95% CI = 98 to 99), for influenza B. Our results support the concept of app-supported, prepositioned at-home RDT kits using symptom-based triggers, although it cannot be recommended with the RDT used in this study. Further research is needed to determine ways to improve the accuracy and utility of home-based testing for influenza.

## INTRODUCTION

Respiratory viral infections (RVI) pose a challenge to clinicians and public health due to their high transmissibility and rapid onset of symptoms, which together cause a high burden of disease (1–4). Development of rapid diagnostic tests (RDTs) for use at point of care (POC) will support clinicians in diagnosing RVI promptly, which can inform actions such as prescribing antivirals (5–7) and interventions to curb transmission (8, 9). While multiple RDTs are approved for use in clinical settings, the COVID-19 pandemic has accelerated efforts to develop RDTs for at-home use (10). At-home RDTs provide an option for sick patients to test without exposing nurses, doctors, or other patients at clinical testing sites, convenience for individuals, including parents, who may be unable to find a window in which to schedule or attend an in-person appointment, and they make it easier for high-risk individuals, such as the elderly or immunocompromised, to test without risking exposure. At-home RDTs for single or combinations of respiratory pathogens such as influenza, respiratory syncytial virus (RSV), and SARS-CoV-2, will likely become prominent public health tools by empowering individuals with accessible patient-led testing after the pandemic has ended.

The most cost-effective and simple RDTs are lateral flow immunochromatographic assays that detect viral antigen from patient samples. Several lateral flow tests (LFT) exist for influenza detection, and several have been recently authorized for detecting SARS-CoV-2 antigens in multiple countries (11–16). Studies describing the accuracy of LFTs for influenza have shown variable sensitivities between 19% and 96% but high specificities in the range of 97% to 99% when used in ambulatory care settings (17–20). Lower sensitivity of LFTs used in ambulatory or home settings is not limited to influenza detection; several studies now demonstrate variable and, in some cases, suboptimal sensitivity of antigen-based tests for SARS-CoV-2, RSV, and human metapneumovirus (21–25).

One of the major methodological challenges in evaluating the accuracy of home-use tests for influenza (and other RVI) is the often-rapid decrease in viral material present in the upper respiratory tract (26, 27). Low viral load may be responsible for the poor sensitivity of antigen-based tests when they are conducted several days after illness onset. To evaluate the impact of minimizing this time interval on the sensitivity of influenza home tests, we employed a novel study design involving prepositioned lateral flow influenza tests and reference swabs with participants before influenza season. Over a period of 4 months, individuals self-reported symptoms in a daily survey which prompted the use of an influenza RDT and self-collected sample for reference testing. We present the successes and weaknesses of this novel study design in relation to the accuracy of the influenza RDT compared with the reference test.

## MATERIALS AND METHODS

### Study design.

This prospective, observational cohort study used a non-probability-based (convenience) sample of participants who were recruited from existing users of the Achievement platform, an online health research community that uses a points-based system to reward participation in studies and has more than 4 million active users (myachievement.com, Evidation Health Inc., San Mateo, CA). This study was part of a broader study conducted by Evidation Health, known as the Home Testing of Respiratory Illness Study, or the “Homekit2020” study, which had a broader goal of developing a methodology to better classify and detect influenza cases and other RVIs through the use of behavioral data collected via wearable devices and patient-reported outcomes (https://www.synapse.org/#!Synapse:syn22803188/wiki/606343). The study was approved by the Western Institutional Review Board (WIRB, Puyallup, WA, USA) and the University of Washington IRB (Study # 1271380). Reporting of this study adheres to the Standards for Reporting of Diagnostic Accuracy Studies (STARD) guidance (28).

### Sample size.

Due to the exploratory nature of this study, sample size was not based on a formal calculation. We aimed to enroll 4,900 participants of whom 175 people were estimated to have confirmed influenza infection, assuming a 75% compliance rate and a national influenza incidence rate of 7% to 10% consistent with previous similar studies (29, 30).

### Participant recruitment and enrollment.

Email invitations were sent to approximately 50,000 users of the Achievement platform between December 13, 2019 and January 31, 2020. Eligible participants were 18 years or older, lived in the United States, could read and understand English, and owned a Fitbit they were willing to wear day and night for the study duration. Participants also agreed to respond to short daily health surveys and were required to have an iOS or Android smartphone or tablet capable of supporting the flu@home app (Audere, Seattle WA, USA). Participants were required to wear a Fitbit for the duration of this study; analyses of Fitbit data are not presented in this manuscript. After confirming eligibility and consenting, participants completed a baseline survey of demographics, history of respiratory illnesses, medication usage, influenza vaccination, and quality of life. Participants who completed all these steps were considered enrolled.

### Flu@home test kit.

Enrolled participants were mailed a flu@home kit after their enrollment was confirmed (Appendix S1 in supplemental materials). Kits contained a QuickVue Influenza A+B test strip (henceforth referred to as the index test), reference sample collection materials, and mailing materials. In addition to the test strip, index test materials consisted of a QuickVue foam-tipped swab, QuickVue reagent tube, and saline ampule (Quidel Corporation, San Diego, CA, USA). Reference materials included an additional QuickVue swab and a tube containing Universal Viral Transport System (UVT) (BD Co., Franklin Lakes, NJ, USA).

### Influenza-like illness monitoring and flu@home kit trigger.

Daily email surveys asked participants if they were currently experiencing any influenza-like illness (ILI) symptoms (defined as cough, fever, chills, sweats, or aches). Those who responded affirmatively were asked additional questions about the presence and severity of their symptoms, medication usage, and quality of life. Participants who reported cough (of any severity) along with aches, fever, chills, and/or sweats (of any severity) met the study trigger criteria and were prompted to download the flu@home app and complete testing procedures. If participants did not experience ILI, additional data were collected consisting of a short survey about their physical, emotional, and symptom status for the last 24 h (Appendix S2). These data were collected as part of the broader Homekit2020 study and are not presented in this comparative accuracy paper. Participants who finished testing procedures were sent a short recovery survey a few weeks later (Appendix S2) asking about actions taken to seek care, medications prescribed by health professionals, illness recovery time, and experience using the flu@home app.

### Index test and reference swab.

The flu@home app guided participants to self-collect an anterior nares (nasal) swab, first for the index test procedures and then for reference sample collection (Appendix S3). Nasal swabs were used because it would not be safe or feasible for a participant to self-collect a nasopharyngeal swab (NPS), and because there is evidence supporting the use of self-collected nasal swabs for detection of influenza (31, 32). Instructions in the mobile app were the same for both the index test and the reference swab and were similar to those in the QuickVue package insert. For both samples, participants were instructed by the app to insert a foam-tipped swab approximately one half inch into each nostril and rotate four times (per nostril) while maintaining contact with the inner wall of the nostril, as demonstrated by an animation in the app. A 10-min in-app timer tracked index test processing time during which participants completed an illness survey (Appendix S4). After index test processing was complete, participants responded to an in-app survey denoting the presence/absence of test and control lines on their index test and followed instructions to take a photo of their index test (Appendix S3). Test strip images were uploaded to a database managed by the study team and were reviewed by a trained “expert” interpreter who determined whether test and control lines were present or absent using the same prompts as the participant, with the addition of an “uninterpretable” option (for instance, due to poor image quality). Neither the expert nor participants knew the outcome of the reference test when evaluating index test results. Index and reference test results were not communicated to participants.

### Reference testing.

Reference samples were shipped to Molecular Testing Labs (MTL, Vancouver, WA). Samples with sufficient material were either tested immediately or frozen at −80°C for later testing. Temperature of the sample during transit was monitored using a TransTracker CF card (Temptime Corp., 116 The American Road, Morris Plains, NJ) capable of recording a single temperature excursion below 0°C and/or a single excursion above 25°C. All samples were tested, regardless of temperature excursions, on the Panther Fusion system according to the manufacturer’s instructions (Hologic inc., San Diego, CA, Cat. No. 303095). In the lab, 0.5-mL aliquots of each sample were extracted and eluted using Panther Fusion buffers (Hologic #PRD-04331, Hologic #PRD-04334), and purified total nucleic acids were tested using multiplex reverse-transcription PCR (RT-PCR) for influenza A, influenza B, and RSV (Hologic #PRD-04328). RT-PCR cycle thresholds (Ct) were used as an indicator of relative viral load. Ct is the number of PCR cycles counted before the signal exceeds the detection threshold; it is inversely related to the viral load in the sample. Generally, genetic material doubles during each cycle of PCR and thus one cycle corresponds to an approximate 2-fold change in nucleic acid concentration.

Reference test results were linked to participant records using a unique barcode. Lab personnel did not have access to index test results or to any participant data. Reference test results were considered positive if the Panther Fusion detected a signal for the specific pathogen and the internal control results were valid for that run.

### Analysis.

All analyses were conducted using R v4.0.2 and RStudio v1.3.1056 (30, 33). Descriptive statistics of the study population and univariate analysis were conducted for demographics, symptom presence and severity, comorbidities, and risk factors. Results were stratified by reference test result and compared using Pearson’s chi-square with Yates' continuity correction, or Fisher’s exact test where appropriate. Illness duration was measured by two methods: (i) trigger-test interval, calculated as the time interval between submitting the survey that triggered testing and the date/time when the index test was performed; and (ii) symptom onset-test interval, calculated as the time interval between the self-reported date of illness onset and the date/time the index test was performed.

Inter-rater reliability between user and expert interpretation of the index test was measured with Cohen’s kappa. Sensitivity, specificity, and 95% confidence intervals (CI) were calculated using the *TableOne* package (34). Performance of the index test was determined for participant interpretation of the index test and for expert interpretation of index test photos, for detection of influenza A and B independently, and for the overall ability to detect influenza regardless of strain. For the latter, participant and expert results were considered “influenza positive” if the index test was interpreted as either influenza A or B positive, “influenza negative” if negative for both, and “invalid” if they indicated no control line. The QuickVue instructions recommend retesting if the RDT is positive for both influenza A and B. Because only one RDT was provided to each participant, retesting was not feasible. Therefore, we considered the RDT invalid if the participant indicated a dual positive and either the expert read or the PCR result disagreed with the dual positive result (i.e., if the expert saw one or zero test lines in the photo or if the PCR result was not a dual positive). All eight index tests marked positive for both influenza A and B were determined to be invalid under these criteria. Tests marked invalid by the participant or by the expert were excluded from their respective analyses.

Comparisons between mean PCR Ct values were performed using a two-tailed, paired Student’s *t* test. Correlation between Ct and number of symptoms was calculated using Pearson’s r (r) and between Ct and reported impact on activities using Spearman’s rho (r_s_). Linear models for symptom trigger-test, symptom onset-test times and number of symptoms, each as a function of Ct, were fit using the “lm” function of the *stats* base package. Models fit to trigger-test intervals, onset-test intervals, and number of symptoms were controlled for age and for each other. Smoothed fits for Ct values as a function of trigger-test and onset-test were done using the “geom_smooth” function in the *ggplot2* package, utilizing a local regression (LOESS) method. All visualizations were created using *ggplot2* (35).

### Data availability.

Study data are available for validated users of Synapse, which is a findable, accessible, interoperable, reusable (FAIR), and HIPAA-compliant platform managed by Sage Bionetworks (https://www.synapse.org/#!Synapse:syn22803188/wiki/606343; Synapse ID: syn22803188).

## RESULTS

### Study completion and participant demographics.

**FIG 1:**
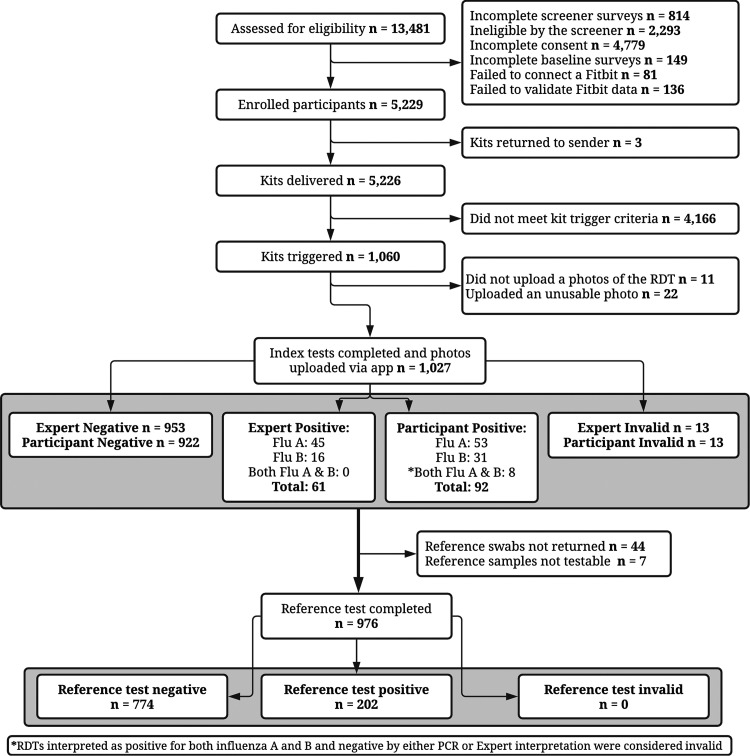
Participant flow.

Between mid-December 2019 and early February 2020, a total of 5,229 individuals met inclusion criteria and were enrolled (Fig. 1). A total of 1,060 participants experienced the symptoms required to trigger their flu kit, of whom 1,027 (97%) completed their index test and uploaded an interpretable index test image. Of those, 976 (95%) also returned a reference swab of which 247 (25.3%) experienced a freezing temperature excursion during transportation and 12 (1.2%) a heating temperature excursion; all samples were tested regardless of temperature excursion and the 12 that experienced a heat excursion were negative by PCR and their associated index tests were also negative. All 976 participants who returned a reference swab also completed the illness survey in the flu@home app; analyses are based on this population.

Participants from all 50 states and the District of Columbia enrolled (median 13 participants per state, IQR: 7 to 25) (Appendix S5). Median age was 36, and participants were predominantly white, college-educated, female, and in the middle- and upper-income tiers (Table 1). Most (94%) reported having health insurance, half (50.7%) received the 2019–2020 flu vaccine, and 694 (71%) reported having at least one comorbidity. More than 99% (968/976) of participants who finished testing procedures also completed the follow-up survey 2 to 3 weeks later. Overall, participants strongly agreed that the instructions in the flu@home app were clear and helpful (796/968, 82%), the app was easy to use (749/968, 77%), and a slightly smaller proportion strongly agreed that the two flu@home nasal swabs were easy (639/968, 66%) (Appendix S6). This rating largely held across age groups, education, and whether or not the participant’s reference test was positive or negative.

**TABLE 1:** Study population characteristics

	Overall (976)	Influenza negative (774)	Influenza positive (202)	*P*
	*N* (%)			
Sex				0.107
Female	759 (77.8)	612 (79.1)	147 (72.8)	
Nonbinary	2 (0.2)	2 (0.3)	0 (0.0)	
Race/Ethnicity*				0.014
Hispanic	60 (6.1)	46 (5.9)	14 (6.9)	
White	901 (92.3)	717 (92.6)	184 (91.1)	
Black/African American	41 (4.2)	30 (3.9)	11 (5.4)	
Asian	43 (4.4)	35 (4.5)	8 (4.0)	
American Indian, Alaska Native, Hawaiian, or Pacific Islander	16 (1.6)	12 (1.7)	3 (1.5)	
Other	12 (1.2)	11 (1.4)	1 (0.5)	
Median age [IQR]	36.00 [31.00, 43.00]	36.00 [31.00, 42.00]	36.00 [31.00, 44.00]	0.446
Education				0.535
HS, GED, or less	46 (4.7)	35 (4.5)	11 (5.4)	
Some college	206 (21.1)	170 (22.0)	36 (17.8)	
Bachelors or equivalent	484 (49.6)	373 (48.2)	111 (55.0)	
Graduate/Masters/Doctorate	236 (24.2)	193 (24.9)	43 (21.3)	
No answer	4 (0.4)	3 (0.4)	1 (0.5)	
Income				0.920
<35k	157 (16.1)	124 (16.0)	33 (16.3)	
35k to 50k	150 (15.4)	121 (15.6)	29 (14.4)	
50k to 75k	227 (23.3)	179 (23.1)	48 (23.8)	
75k to 100k	154 (15.8)	120 (15.5)	34 (16.8)	
100k+	241 (24.7)	195 (25.2)	46 (22.8)	
No answer	47 (4.8)	35 (4.5)	12 (5.9)	
Child 0 to 5 in daycare or preschool**	160 (61.3^α^)	119 (57.5)	41 (75.9)	0.020
Received 20192020 flu vaccine	500 (51.2)	399 (51.6)	101 (50)	0.754
Known flu diagnosis in household				0.035
No	820 (84.0)	661 (85.4)	159 (78.7)	
Yes	50 (5.1)	32 (4.1)	18 (8.9)	
Did not know	10 (1.0)	8 (1.0)	2 (1.0)	
No answer	96 (9.8)	73 (9.4)	23 (11.4)	
Suspected exposure to flu				<0.001
No	180 (18.4)	163 (21.1)	17 (8.4)	
Yes	391 (40.1)	283 (36.6)	108 (53.5)	
Did not know	404 (41.4)	327 (42.2)	77 (38.1)	
No answer	1 (0.1)	1 (0.1)	0 (0.0)	
Reports at least one comorbidity	694 (71.1)	550 (71.1)	144 (71.3)	1

*Totals may be greater than total sample size as multi-select was offered; **261 participants reported a 0 to 5-year-old in the household, of which 207 were negative for influenza and 54 were positive for influenza.

### Reference testing and symptoms.

A total of 202 (20.7%) individuals were positive for influenza per the reference test. An additional 21 (2.2%) tested positive for only RSV. Race/ethnicity (*P = *0.014), physician diagnosed influenza in the household (*P = *0.035) and suspected influenza exposure (*P* <0.001) were associated with a positive reference test for influenza (Table 1), while primary state of residence (*P = *0.229) (Appendix S5) and vaccination status (*P = *0.754) (Table 1) were not. The most common symptoms among all participants were cough (80.8%), sore throat (74.3%), fatigue (80.3%), and headache (68.9%) (Table 2). Participants with a positive reference test were more likely to have fever (*P < *0.001), chills (*P < *0.001), sweats (*P < *0.001), aches (*P < *0.001), cough (*P = *0.002), difficulty breathing (*P = *0.031), and vomiting (*P = *0.033) than those with a negative reference test. Individuals with a positive reference test were also more likely to report that their illness felt worse than a typical cold (*P* <0.001), that they thought their illness was the flu rather than a cold or another illness (*P < *0.001), that their symptoms started within the last 1 or 1.5 days (*P = *0.044), and that their symptoms had a greater versus lesser impact on their daily activities (*P < *0.001) than those with a negative test. With a few exceptions, more severe symptoms were associated with a positive influenza PCR test result (Appendix S7).

**TABLE 2:** Symptom presence, symptom onset, and impact on activities in participants with and without lab-confirmed influenza

	Overall (976)	Influenza negative (774)	Influenza positive (202)	*P*
New or worsening:	*N* (%)			
Fever	441 (45.2)	304 (39.3)	137 (67.8)	<0.001
Headache	672 (68.9)	522 (67.4)	150 (74.3)	0.075
Cough	789 (80.8)	610 (78.8)	179 (88.6)	0.002
Chills/shivering	402 (41.2)	270 (34.9)	132 (65.3)	<0.001
Sweats	352 (36.1)	250 (32.3)	102 (50.5)	<0.001
Sore throat	725 (74.3)	569 (73.5)	156 (77.2)	0.325
Vomiting	153 (15.7)	111 (14.3)	42 (20.8)	0.033
Runny nose	741 (75.9)	581 (75.1)	160 (79.2)	0.257
Sneezing	577 (59.1)	466 (60.2)	111 (55.0)	0.203
Fatigue	784 (80.3)	617 (79.7)	167 (82.7)	0.40
Muscle or body aches	597 (61.2)	447 (57.8)	150 (74.3)	<0.001
Trouble breathing	313 (32.1)	235 (30.4)	78 (38.6)	0.031
No symptoms	38 (3.9)	34 (4.4)	4 (2.0)	0.169
Illness felt worse than a typical cold	569 (58.3)	410 (53)	159 (78.7)	<0.001
Participant perceived illness as:				<0.001
Cold	524 (53.7)	459 (59.3)	65 (32.2)	
Flu	314 (32.2)	190 (24.5)	124 (61.4)	
Other	138 (14.1)	125 (16.1)	13 (6.4)	
Symptom onset within:				0.044
0.5 days	0 (0.0)	0 (0.0)	0 (0.0)	
1 day	372 (38.1)	289 (37.3)	83 (41.1)	
1.5 days	161 (16.5)	122 (15.8)	39 (19.3)	
2 days	96 (9.8)	85 (11.0)	11 (5.4)	
3 days	49 (5.0)	44 (5.7)	5 (2.5)	
4 days	10 (1.0)	8 (1.0)	2 (1.0)	
5 days	19 (1.9)	17 (2.2)	2 (1.0)	
No answer	269 (27.6)	209 (27.0)	60 (29.7)	
Activities impacted by illness:				<0.001
Not at all	85 (8.7)	78 (10.1)	7 (3.5)	
A little bit	334 (34.2)	294 (38.0)	40 (19.8)	
Somewhat	282 (28.9)	234 (30.2)	48 (23.8)	
Quite a bit	183 (18.8)	121 (15.6)	62 (30.7)	
Very much	92 (9.4)	47 (6.1)	45 (22.3)	

### Performance of the RDT.

Participants and experts agreed on 932 RDT results (95.5%, kappa = 0.72). Of the 44 tests with conflicting interpretations, 36 were errors made by the participant, including 15 instances where a participant incorrectly reported the presence of a control line and one test line (positive for *either* influenza A or B) when both the PCR results and expert interpretation of the RDT photo were negative (Appendix S8) and eight instances where the participant reported two test lines (positive for *both* influenza A and B) when the expert did not see any test lines (influenza negative) or failed to see a control line (invalid). Additionally, the expert identified four positive tests that a participant marked as negative. When interpreted by participants, the RDT had a sensitivity of 28% (95% CI = 21 to 36) for influenza A and 32% (95% CI = 20 to 46) for influenza B, with specificities for both A and B types of 99% (95% CI = 98 to 99). Participants’ interpretation of the RDT for the overall presence of influenza (either A or B) had a sensitivity of 32% (95% CI = 26 to 39) and a specificity of 98% (95% CI = 97 to 99). There were no significant differences in RDT performance when interpreted by the expert or based on vaccination status (Table 3). The mean Ct value of reference samples was 27.2 cycles (SD = 5.16, IQR = 23.7 to 30.2) and was significantly lower in samples with a true positive (TP) index test (mean = 23.3 cycles, SD = 3.38, IQR = 20.5 to 25.6) than those with a false negative (FN) index test (mean = 29.1 cycles, SD = 4.8, IQR = 25.4 to 32.7) (*P < *0.001).

**TABLE 3:** Accuracy of influenza self-test compared to laboratory reference test

Interpreter	Mean Ct value	TP^*d*^	FP^*d*^	FN^*d*^	TN^*d*^	Sensitivity (95% CI)	Specificity (95% CI)	Positive likelihood ratio (95% CI)	Negative likelihood ratio (95% CI)
Participant^*a*^^,^^*b*^	27.2	64	16	135	743	32% (26 to 39)	98% (97 to 99)	15.3 (9.0 to 25.8)	0.69 (0.63 to 0.76)
Expert	27.2	57	3	144	762	28% (22 to 35)	100% (99 to 100)	72.31 (22.9 to 230)	0.72 (0.66 to 0.78)
Consensus^*c*^	27.3	52	1	131	741	28% (22 to 36)	100% (99 to 100)	210.8 (29.3 to 1500)	0.72 (0.65 to 0.79)
Participant interpretation of flu A^*b*^	27.0	40	11	103	804	28% (21 to 36)	99% (98 to 99)	20.7 (10.9 to 39.4)	0.74 (0.67 to 0.81)
Participant interpretation of flu B^*b*^	27.8	18	11	38	891	32% (20 to 46)	99% (98 to 99)	26.9 (13.4 to 54.1)	0.69 (0.57 to 0.82)
Participant interpretation,^*a*^^,^^*b*^ 20192020 flu vaccine = “Yes”	26.4	30	12	69	380	30% (21 to 40)	97% (95 to 98)	9.90 (5.26, 18.63)	0.72 (0.63 to 0.82)
Participant interpretation,^*a*^^,^^*b*^ 20192020 flu vaccine = “No”	28	34	4	66	363	34% (25 to 44)	99% (97 to 100)	31.2 (11.3 to 85.8)	0.67 (0.58 to 0.77)
Expert interpretation,^*a*^ 20192020 flu vaccine = “Yes”	26.4	30	2	71	392	30% (21 to 40)	99% (98 to 100)	58.5 (14.2 to 240.8)	0.71 (0.62 to 0.80)
Expert interpretation,^*a*^ 20192020 flu vaccine = “No”	28	27	1	73	370	27% (19 to 37)	100% (99 to 100)	100.2 (13.8 to 728.2)	0.73 (0.65 to 0.82)

a Ten tests were designated invalid by participants and by the expert and thus excluded from performance.

b Participants interpreted eight tests as having lines both above and below the control line and these results were excluded from performance analysis.

c Includes only RDTs for which the participant and expert agreed on the result.

d TP, true positive; FP, false positive; FN, false negative; TN, true negative.

**FIG 2:**
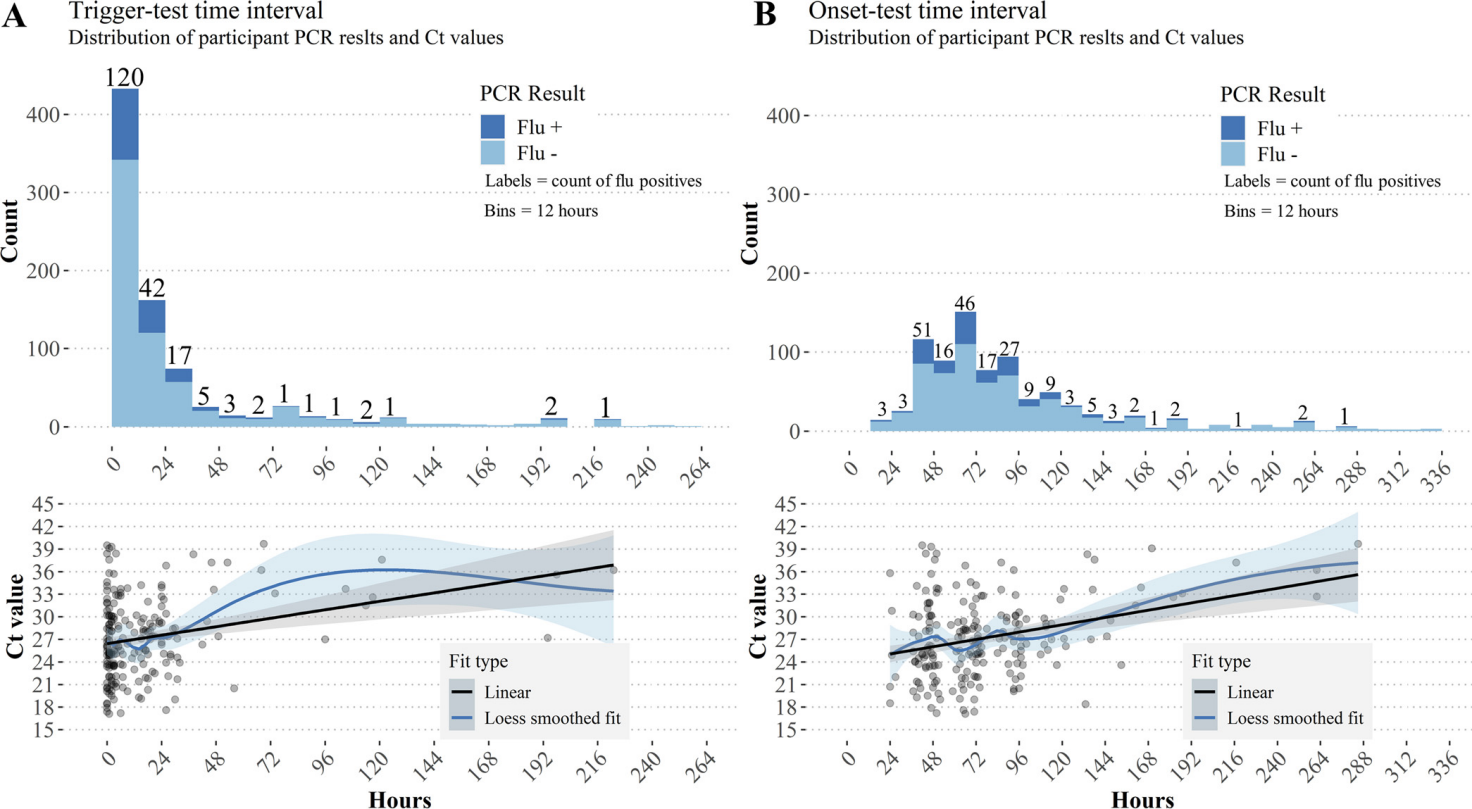
Flu positivity and Ct trends for trigger-test and onset-test intervals. Ribbons indicate 95% confidence intervals for fitted lines. (A) Flu positivity rates decreased as the time between triggering the flu@home kit and using the RDT increased. Though samples were small for longer intervals, there was a linear relationship between Ct and trigger-test interval (*P* < 0.001). B) Time intervals between participant-reported onset of first symptoms and using the RDT were distributed more widely than trigger-test intervals, but positive tests are still concentrated within the first 72 h to 96 h. There was a linear relationship between onset-test intervals and Ct values (*P* < 0.001).

Most participants (714, 73%) completed their index test within 24 h of the trigger, with a median trigger-test interval of 7 h (IQR 2 h to 24 h) (Fig. 2a). In comparison, symptom onset-test intervals were longer and more widely distributed (median = 72 h, IQR = 49 h to 110 h) (Fig. 2b). As both intervals increased, influenza-positive cases declined, and longer intervals were associated with linearly increasing Ct values that suggests an exponential decay of viral load (Fig. 2a and b). Ct values increased by 1.13 cycles (an approximately 2-fold decrease in viral load) per day from trigger (*P* <0.001) and 0.65 cycles (∼1.5-fold decrease in viral load) per day from onset (*P* <0.001) when fit to linear models. However, accuracy of the RDT was not significantly different in sub-analyses that explored performance at longer trigger-test and onset-test intervals (Table 4).

**TABLE 4:** Accuracy of participant-interpreted influenza self-test based on number of symptoms, impact on activities, and time intervals

	Mean Ct value	TP^*b*^	FP^*b*^	FN^*b*^	TN^*b*^	Sensitivity (95% CI)	Specificity (95% CI)
No. of symptoms							
0 to 3	30.1	3	0	10	107	23% (5 to 54)	100% (97 to 100)
4 to 7	28	15	11	55	349	21% (13 to 33)	97% (95 to 98)
8 to 12	26.4	46	5	70	287	40% (31 to 49)	98% ( 96 to 99)
Impact on activities							
Not at all	30	1	0	6	76	14% (0 to 58)	100% (95 to 100)
A little bit	29.1	10	6	29	284	26% (13 to 42)	98% (96 to 99)
Somewhat	27.1	15	4	33	226	31% (19 to 46)	98% (96 to 100)
Quite a bit	26.8	20	6	41	111	33% (21 to 46)	95% (89 to 98)
Very much	25.8	18	0	26	46	41% (26 to 57)	100% (92 to 100)
Trigger-test interval							
0 to 24	26.6	54	10	104	533	34% (27 to 42)	98% (97 to 99)
24 to 48	28.2	9	2	18	83	33% (17 to 54)	98% (92 to 100)
48 to 72	32.2	1	0	4	21	20% (1 to 72)	100% (84 to 100)
72 to 96	33.1	0	1	1	37	0% (0 to 97)	97% (86 to 90)
Onset-test interval							
0 to 24^*a*^	n/a	0	0	0	17	NaN (00 to 00)	100% (80 to 100)
24 to 48	27.1	16	3	36	132	31% (19 to 45)	98% (94 to 100)
48 to 72	25.7	22	4	36	200	38% (26 to 52)	98% (95 to 99)
72 to 96	27.3	14	2	33	145	30% (17 to 45)	99% (95 to 100)

a No influenza positive individuals by index test or reference test within 24 h of reported symptom onset.

b TP, true positive; FP, false positive; FN, false negative; TN, true negative.

**FIG 3:**
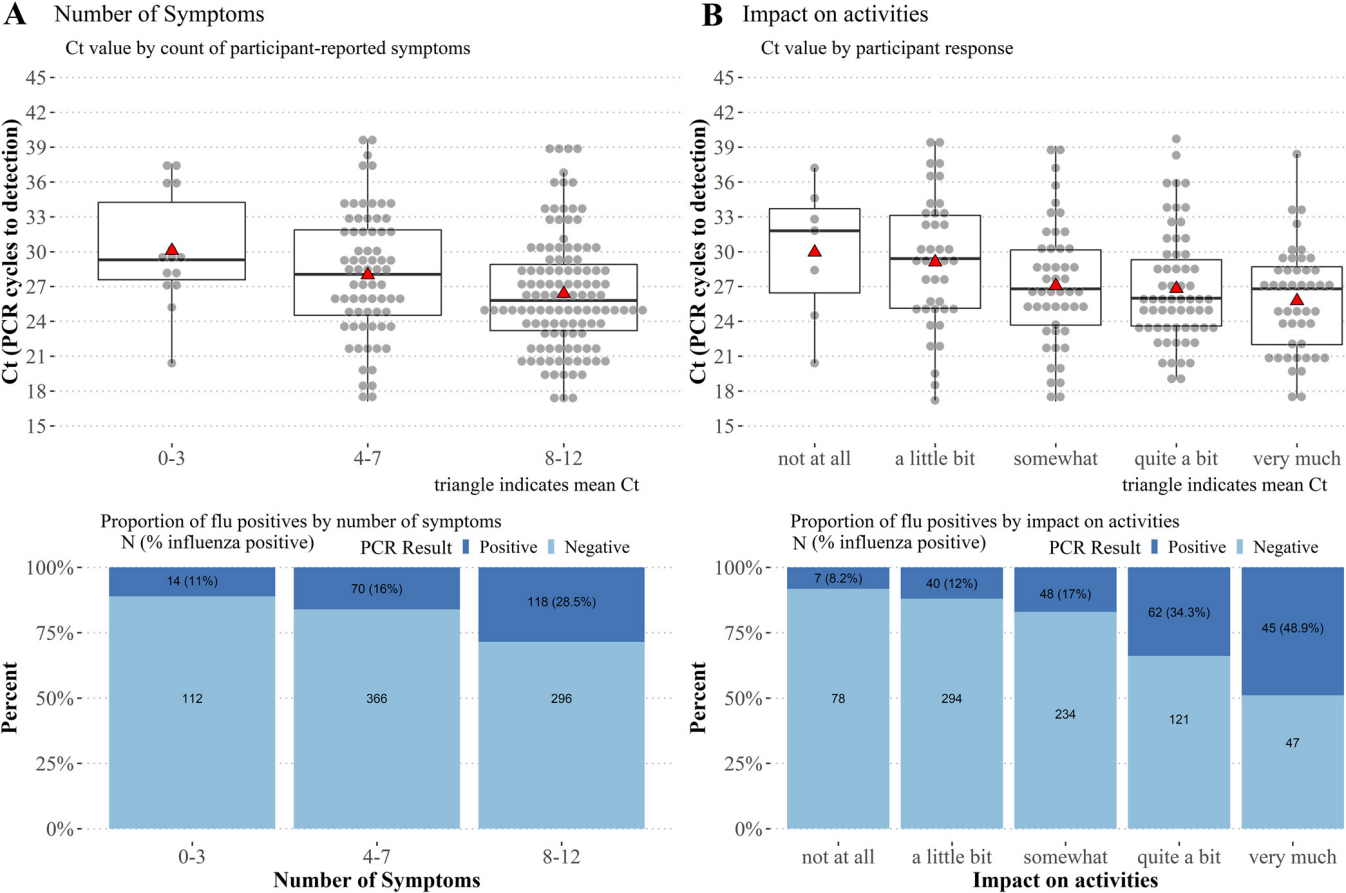
Reference sample Ct trends and proportion of flu positives for number of symptoms and impact of illness on daily activities. (A) Ct values were inversely related to number of self-reported symptoms (*P* < 0.001) and participants with the most symptoms had a higher proportion of positive influenza cases (χ^2^ = 28.15, *P* < 0.001). (B) Participants reported how much their illness impacted their ability to complete their daily activities. Ct values were negatively correlated with reported impact on activities (r_s_ = 0.22, *P = *0.002) and there was a significantly greater proportion of influenza positive cases in the group reporting that their illness “very much” impacted their daily activities (χ^2^ = 89.8, *P* < 0.001).

In addition to time intervals, Ct values were negatively correlated with symptom count (r = 0.27, *P < *0.001), and a linear model of this relationship showed a decrease of 0.36 cycles per each additional symptom (*P = *0.006). This model also showed an interaction with days from onset (*P = *0.015). When grouped by 0 to 3, 4 to 7, and 8 to 12 symptoms, mean Ct values were significantly lower for the 8 to 12 group than when compared with the 0 to 3 group (Tukey’s test, *P* = 0.028) but not the 4 to 7 group, and there was a higher proportion of flu positives in the subgroup with the most (8 to 12) symptoms (χ^2^ = 28.15, *P* < 0.001) (Fig. 3a). Sensitivity of the index test increased from 21% (95% CI = 13 to 33) to 41% (95% CI = 32 to 50) for ≥ 8 symptoms (Table 4). Ct values were negatively correlated with impact of the illness on participants’ ability to conduct their daily activities (r_s_ = 0.22, *P = *0.002) and there was a higher proportion of flu positive participants reporting a higher impact on activities (χ^2^ = 89.8, *P* < 0.001) (Fig. 3B). RDT sensitivity increased with greater impact on daily activities, though confidence intervals were overlapping (Table 4).

## DISCUSSION

### Main findings.

Early identification of individuals with respiratory symptoms using testing to confirm infection are key steps to clinical management of RVI such as influenza and SARS-CoV-2 (5, 6, 36, 37). By prepositioning test kits and identifying ILI through daily symptom surveys we were able to minimize time intervals from onset of specific symptoms to RDT use. Most participants (714, 73%) used their test kit within 24 h of triggering it, and half within 7 h (496, 51%). Low sensitivity of the QuickVue antigen based RDT was similar to that found in other studies (17, 18, 20), suggesting that testing shortly after onset of the influenza-specific symptoms selected as trigger criteria did not improve the poor limit of detection of this assay. Our findings do not support using the QuickVue A+B RDT for at-home diagnosis of influenza (note, that QuickVue is a CLIA-waived test and was not approved for at-home use).

We hypothesized that earlier testing may improve sensitivity of the QuickVue RDT based on the premise that peak influenza viral shedding occurs 48 h after infection and 24 h before the most severe symptoms appear, then declines rapidly (38). One possible explanation for the poor sensitivity even at the earliest trigger-test and onset-test intervals is that our selected trigger criteria still missed the window when viral load was highest. While the study protocol successfully prompted testing within a few hours of the trigger (short trigger-test intervals), there was a considerably longer time between the date the participant retrospectively reported first feeling sick and when they tested (long symptom onset-test intervals), typically about 72 h. The trigger in our study was contingent upon the presence of cough and thus participants likely experienced symptoms both specific and nonspecific to influenza before triggering their kit. For an antigen based RDT such as the QuickVue, it is possible that trigger criteria prompting even earlier testing, possibly followed by serial testing, may increase the likelihood that the test is conducted within the window of peak viral shedding, when the sensitivity of the RDT will be maximized. This could be accomplished by relaxing the requirement for cough to be present and by including more general RVI symptoms in the criteria. However, this type of approach would have to be weighed against the potential costs of a greater number of tests needed at a population level and a potentially higher false positive rate if influenza prevalence is low. Conversely, test sensitivity was better for participants reporting a greater number of symptoms or impact on activities, which could potentially be valuable (in addition to, or instead of) the criteria we used to optimize test performance for an at-home influenza RDT.

To further understand what participant-reported factors could be related to periods of high viral load, we examined test performance, influenza infection rate, and Ct value for two additional measures: number of symptoms reported and perceived impact of illness on daily activities. Participants who reported the most symptoms and the greatest impact on their daily activities had lower Ct values and higher RDT sensitivity. Furthermore, participants who reported that they felt worse than when they have a typical cold were more likely to have influenza, as were participants who thought they were sick with influenza rather than a cold or another illness. Together, this evidence suggests that objective presentation (such as number of symptoms) and the participant’s subjective perception of illness might serve as effective guides for when to test or provide additional credence to test results. Indeed, several clinical prediction rules (CPRs) have been shown to be moderately effective at predicting influenza on their own, with high-risk likelihood ratios (LR) in the range of 4 to 7.8 and a low-risk LR of 0.06 to 0.72 (39, 40), comparable with the positive and negative LRs found for the user-interpreted QuickVue test in this study (15.3 and 0.69, respectively). CPRs, however, face the challenge of distinguishing influenza from other ILI such as RSV and COVID-19 (41–43), preserving the crucial role of diagnostics in confirming the causative pathogen. An additional, promising, source of trigger criteria that is actively being researched is person-generated health data gathered by consumer wearable sensors. The physiological measures that these sensors capture, such as resting heart rate, change in activity level, or peripheral oxygen saturation (SpO_2_), etc., could provide indicators that a person is unwell before they are aware of any symptoms and may provide a method to differentiate between RVIs with symptom overlap, such as influenza and COVID-19 (44, 46, 47).

### Comparisons to other studies.

Previous studies have reported uniform high specificity of the QuickVue A+B test and highly variable sensitivities ranging from 19% to 96% (17–20, 48–50). Studies conducted at inpatient or hospital settings tend to find higher sensitivity of the RDT, which may be due to more severe infection and higher viral load (38, 51), or the use of pooled nasal and throat swabs or nasopharyngeal aspirate (50) compared with studies in community settings that used nasal swabs (18), as ours did. Other studies have found higher viral load and higher sensitivity of the QuickVue RDT in children (48, 50), raising the possibility that the low sensitivity found in our study may be due in part to an overall lower viral load in our adult, less-severely-ill, at-home population.

To our knowledge, only three peer-reviewed studies (52–54), including a pilot of the flu@home study conducted in 2018, report the performance of an at-home influenza RDT. The pilot flu@home study authors attribute the 14% sensitivity they found for the QuickVue in part due to delays in testing after illness onset—nearly 80% of RDTs were used four or more days after illness onset. The other two studies differ in that they utilized an antigen-based RDT with fluorescent detection that was designed specifically for home-use. These studies report moderate sensitivity (61% and 72.7%) and specificity (95% and 96.2%) of the RDT. While one of them used a regular symptom survey to prompt testing, it was reported weekly, not daily.

### Strengths, limitations, and future directions.

Our study design had several strengths. Using prospective recruitment, we were able to preposition test kits in a large national sample prior to onset of influenza season and used daily symptom surveys over a 4-month period to trigger use of the at-home RDT and reference swabs. This design facilitated testing within just hours of when trigger symptoms were reported, as well as collection of precise time points throughout the study protocol. While our study was conducted during the outset of the COVID-19 pandemic and mitigation efforts could have impacted the prevalence of influenza, the overall influenza burden for the 2019–2020 season was similar to other years (55) and there was a high proportion of individuals with influenza among the participants we tested.

We are aware of several limitations. First, our study population was younger and less diverse in terms of education, income, and race/ethnicity than the U.S. population overall, likely due to the inclusion criteria that participants must own and use a Fitbit and possibly due to the targeted recruitment of individuals who engage with an online health platform. While this potentially limits the generalizability of the results, the population was composed of active users of digital health tools who are likely representative of early adopters of home-health technology such as an at-home RDT. Future studies should use broader recruitment criteria that do not require use of Fitbits and include more generalizable populations. While we did not conduct a cost-effectiveness analysis, only one fifth of the tests sent to participants were used, which amounts to a non-negligible sunk cost in materials and distribution fees. The approach may still be feasible for test developers seeking to validate an at-home diagnostic, and future researchers may be able to devise methods for participants to return unused tests to recover material costs. Left as-is, the expense of the study may pose a barrier to widespread implementation of this model.

Second, we achieved a high study completion rate but there were a number of protocol deviations that could have been avoided. A small number of samples experienced temperatures in excess of 25°C, outside the recommended range of the UVT used in this study. While samples are likely to tolerate freezing excursions, overheating events may impact sample stability. In our case, all “overheated” samples were negative by PCR and by index test, and the number (12) was small enough that at most one false negative result was likely (given an overall FN rate of 7%); however a, higher proportion of heat excursions could have negatively impacted results. Future studies should take into consideration climate and season during which the samples will be shipped or consider using dry swab collection instead of UVT. Dry swabs have been shown to be stable at high and low temperature excursions for SARS-CoV-2 (56, 57) and future studies may show similar stability for influenza. In addition, 51 participants did not return a reference swab, or returned a swab that was unable to be tested. Future studies could address this drop-off by conducting reference sample collection before the index test, and by linking compensation to the confirmed scanning of a shipping label or reception by the post office or courier service.

Third, this study opted to use the QuickVue test, a “subjective read” RDT (srRDT) that requires visual interpretation of the test line. Despite the app-based step-by-step instructions for test interpretation, including photo examples of negative, positive, and invalid test results, participants still made errors when interpreting the RDT at a higher rate than experts, confirming the importance of photos to validate index test results when using a srRDT. Furthermore, some participants failed to upload a picture or uploaded one that was too blurry or dark to be used, resulting in a loss of data. To help mitigate the loss of photos, future iterations of an app could consider automatic flashes, better auto-focusing, local storage of the photo file in the absence of a cellular or Wi-Fi connection, and study kits that include a stand to hold the phone steady and at the appropriate distance. The failure of some participants to correctly interpret this srRDT suggests that other types of digital aids, such as computer vision (CV) assisted interpretation of the RDT, ought to be developed, in addition to the step-by-step instructions for conducting the RDT.

Alternatively, some at-home diagnostics do not require subjective interpretation of the results. Digital RDTs (dRDTs) use electronics in the test cassette or an external instrument to calculate a qualitative positive/negative/invalid result from a chromatographic or fluorescent signal and tend to be more sensitive than srRDTs (58, 59). Home-based molecular RDTs, such as those that have been approved by the FDA for detection of SARS-CoV-2 (11, 12), appear to be highly sensitive and also do not require subjective interpretation of a test result. Both dRDTs designed for at-home use, such as those used by Geyer et al. and Heimonen et al. (53, 54), and molecular RDTs thus present a good option to improve the diagnostic utility of at-home testing. However, subjective read RDTs are considerably less expensive than either of these alternatives and have been widely used during the COVID-19 pandemic for at-home diagnosis. It is crucial to understand the strengths and limitations of this type of assay, particularly when it comes to interpretation of test lines and improving sensitivity. A human-centered assessment of subjective read RDT design could effectively pinpoint weaknesses when the test is operated by a lay user and could inform future design strategies for at-home RDTs (60, 61).

Lastly, we selected only one set of trigger criteria that included symptoms widely cited as ILI. Our results suggest that using earlier signals of illness onset may enable testing when viral load is highest, improving test sensitivity. Future studies should therefore employ other individual test triggers or combinations of test triggers to better understand their performance as trigger criteria and their relation to periods of high viral load. Such criteria could include less-specific symptoms, input from wearable devices, and participant-reported illness experience, such as impact on activities and rapid onset of illness.

### Implications for patients, policymakers, clinicians, and researchers.

Our findings demonstrate that individuals are capable of collecting a nasal sample and performing and interpreting an RDT without clinical oversight when guided by instructions and process controls in a companion app. Enabling easy access to RDTs for RVIs in at-home environments could boost public health efforts by providing individuals with information they can use early in their illness to make the best decisions for their care, including infection control efforts to reduce transmission. Despite the younger and potentially more technologically aware study population and the use of app-guided interpretation of the RDT complete with photo examples of positive, negative, and invalid results, our study did identify a minority of participants (*n* = 36) who misinterpreted the test result, which could lead to incorrect decision making. The impact of these errors, especially “wrong line” errors, could be compounded by the development of at-home RDTs multiplexed to detect influenza and SARS-CoV-2 (62), which could have more lines and may be more complex to interpret. In these cases, digital aides for test interpretation, such as automated CV and artificial intelligence (AI)-driven interpretation of test strips, could provide important adjuncts to support safe use and appropriate follow-up care.

While our study deployed an RDT not approved or authorized for at-home settings, our results suggest that tests of this level of complexity can be completed successfully by individuals when accompanied by digital guidance. Device regulators and clinicians should determine whether additional criteria should be required to recommend use of the test based on its known sensitivity, and whether to impose more stringent criteria or serial testing for less sensitive tests (63). Furthermore, given the possible variation in symptoms associated with different strains of influenza, other RVI, and presence of comorbidities (64–66), a symptom-based approach to triggering of self-tests may be inherently limited. Researchers may leverage study designs similar to this to generate more evidence for the use of physiological markers from wearable sensors or participant-reported factors in guiding at-home test use (47).

By prepositioning app-supported influenza at-home tests at the start of the study and thus empowering individuals to test shortly after the onset of target symptoms, our study provides support for a form of self-testing that could potentially apply not only to influenza, but to other RVI. While our findings do not support the use of the QuickVue A+B RDT for at-home influenza testing, antigen-based immunochromatographic assays remain the most effective means to get diagnostics into at-home settings and are playing a crucial role in the COVID-19 pandemic (67, 68), and thus all potential avenues to improve their performance should continue to be explored. Our results should prompt the exploration of more sophisticated triggers indicating the onset of infection, such as resting heart rate or SpO_2,_ which the participant may not be aware of, but which can be detected by wearable sensors. Our results should also encourage research and innovation in digital aids that help participants execute testing procedures, and future researchers may leverage our study design and lessons learned to serve as a template for studying the performance of at-home test use, for individual care and public health surveillance related to influenza and other RVI.
