# Supplementary material for: Flu@home: the Comparative Accuracy of an At-Home Influenza Rapid Diagnostic Test Using a Prepositioned Test Kit, Mobile App, Mail-in Reference Sample, and Symptom-Based Testing Trigger
Source: J Clin Microbiol. 2022 Mar 16;60(3):e02070-21. doi: 10.1128/jcm.02070-21 (PMC8925896; doi:10.1128/jcm.02070-21)
Supplement: Supplemental file 1 — Appendixes S1 to S8. Download jcm.02070-21-s0001.pdf, PDF file, 1.1 MB [file jcm.02070-21-s0001.pdf]

Appendices

Appendix 1: flu@home kit contents ©2019 Audere (p1)

Appendix 2: Daily Surveys

Appendix 2.1: Daily Questionnaire (p3)

Appendix 2.2: Daily Survey A [participant responded "yes" to daily questionnaire] (p3)

Appendix 2.3 Daily Survey B [participant responded "no" to daily questionnaire] (p12)

Appendix 2.4 – Recovery Survey and flu@home user feedback (p14)

Appendix 3: flu@home app, ©2019 Audere (p22)

Appendix 4: flu@home in-app surveys

Appendix 4.1: Symptom Survey (p24)

Appendix 4.2: flu@home test and control line presence questions (p31)

Appendix 5: Primary state of reference for influenza positive and negative participants (p32)

Appendix 6: Participant responses to flu@home feedback questions

Appendix 7: Severity of individual symptoms in participants with and without influenza confirmed by laboratory testing (p37)

Appendix 8: Disagreement over index test results between participants and experts and count of each error type (p40)

Appendix 1: flu@home kit contents ©2019 Audere

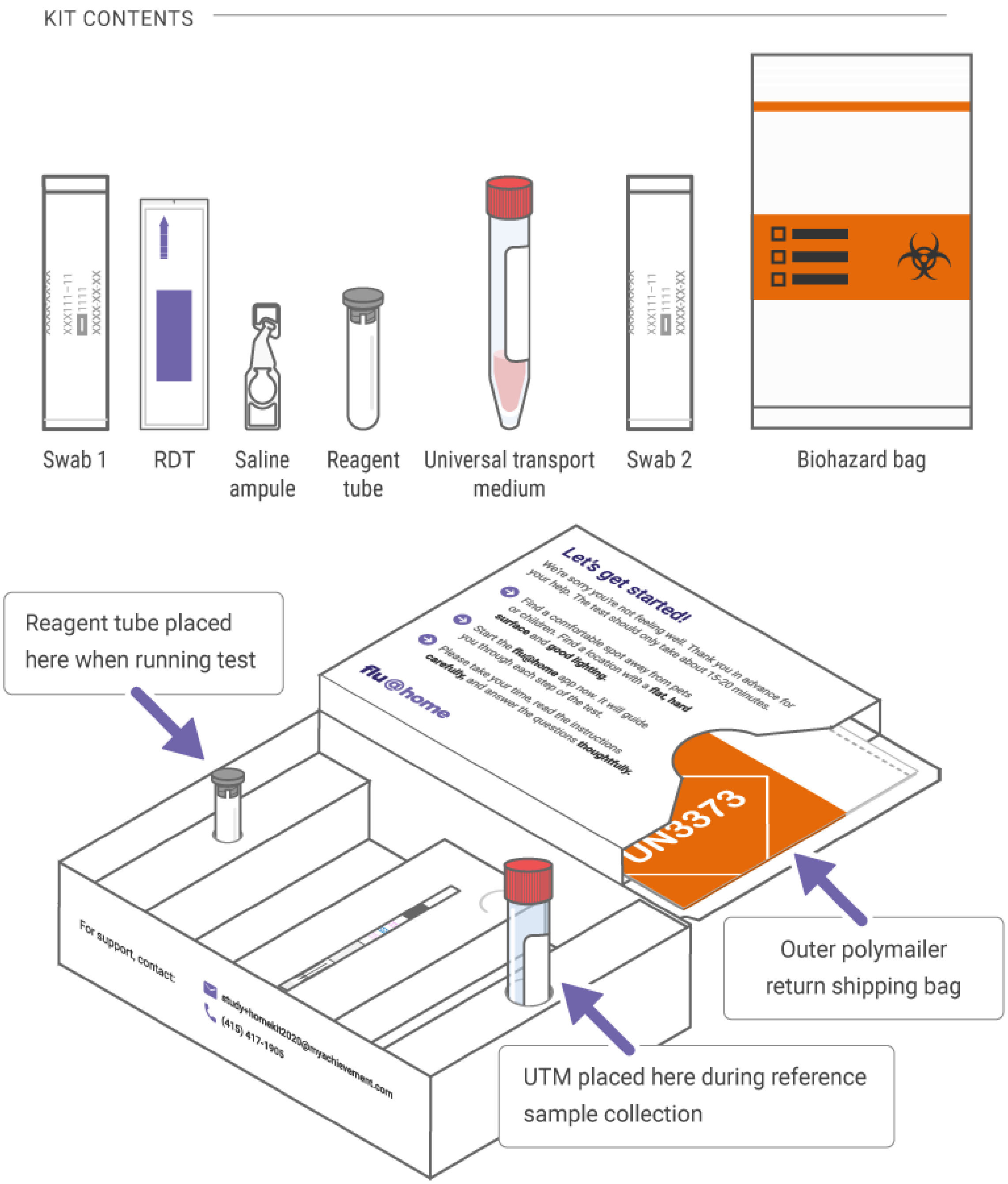

Appendix 2: Daily Surveys

<u>Appendix 2.1: Daily Questionnaire</u>

1. **Have you experienced any flu-like symptoms in the past 24 hours?**
   Such as: Cough, Fever, Chills, Sweats, Body Aches
   a. Yes, I <u>have</u> flu-like symptoms
   b. No, I <u>do not</u> have flu-like symptoms

[IF Q1="YES", THEN USE 'Daily Survey A']

[IF Q1="NO", THEN USE 'Daily Survey B']

<u>Appendix 2.2: Daily Survey A [participant responded "yes" to daily questionnaire]</u>
Please answer the following questions based on your experiences in the past 24 hours.

If you have selected "Yes, I have flu like symptoms" by mistake and have **not** experienced flu-like symptoms in the **past 24 hours**, please do not complete this survey. You can contact study support to report a change to your daily questionnaire response and they will prompt you on next steps.

2. As of this moment, do you feel that you:
   a. Are currently feeling symptoms of the flu
   b. Are fully **recovered**, but felt symptoms of the flu <u>within the last 24 hours</u>

[IF Q2 = "b" feel recovered now, ask Q3 & Q4 then ASK Q6-Q8, then SKIP to Q12]

[If Q2 = "a" feel symptoms of flu, skip to Q5]

Symptoms

3. Please specify the exact date and time that you felt completely recovered from your flu or flu like illness?
   a. [DATE/TIME INTERFACE]

4. Please specify and <u>rate the severity</u> of the flu-like symptoms you were feeling in the **past 24 hours**.
   If you have not experienced one of the symptoms listed in the table within the past 24 hours, please select "None". If you have experienced one of the symptoms listed in the table, please select mild, moderate, or severe based on how you are/were feeling in the past 24 hours.

	None (0)	Mild (1)	Moderate (2)	Severe (3)
Cough				

Body/Muscle aches				
Feeling feverish				
Chills or shivering				
Sweats				
Sore throat or itchy/scratchy throat				
Feeling more tired than usual				
Nasal congestion or runny nose				
Sneezing				

5. Please specify and rate the severity of **any flu-like symptoms** that you are <u>currently experiencing</u> or <u>have experienced</u> in the **past 24 hours**.

   If you have not experienced one of the symptoms listed in the table, please select "None". If you have experienced one of the symptoms listed in the table, please select mild, moderate, or severe based on how you are feeling.

	None (0)	Mild (1)	Moderate (2)	Severe (3)
Cough				
Body/Muscle Aches				
Feeling feverish				
Chills and shivering				
Sweats				

Headache				
Sore throat or itchy/scratchy throat				
Fatigue				
Nasal congestion or runny nose				
Sneezing				

[IF **Q5.a does not equal "None"** (Cough) AND any of the other response options from "Body/Muscle Aches" OR "Feeling feverish" OR "Chills or shivering" OR "Sweats" **does not equal "None"** --> PARTICIPANTS WILL BE PROMPTED TO COMPLETE THEIR AT-HOME FLU TEST KIT & WILL CONTINUE ON IN THE DAILY SURVEY]

6. Is this the first time you are reporting feeling flu-like symptoms in the past 14 days to the daily questionnaires?
   a. This is my **FIRST TIME** reporting flu-like symptoms
   b. I have reported flu like symptoms in a previous daily questionnaire

[If Q6 = a, ASK Q7]

7. When did your flu or flu-like symptom(s) first start?
   a. [DATE PICKER]

8. Please rate the overall severity of your flu or flu-like illness in the **past 24 hours**.
   a. Mild
   b. Moderate
   c. Severe

9. Do you have a thermometer available and are you willing to report your **current body temperature**?
   a. Yes
   b. I prefer not to report
   c.

[IF Q9="YES", THEN ANSWER Q10 & Q11]

[IF Q9 = "I prefer not to report", THEN GO TO Q12]

10. Please record your **current body temperature** in degrees Fahrenheit and include the first decimal value (e.g. 100.4).

a. [FREE TEXT NUMBER ENTRY]

11. Where did you take your temperature?
    a. Forehead
    b. Armpit
    c. Mouth
    d. Ear
    e. Other

Flu Medications

12. Did a medical professional <u>prescribe</u> any **medications** to <u>treat or manage</u> your current flu or flu-like symptoms?
    a. Yes
    b. No
    c. I don't know / I can't remember

[IF Q12="Yes", THEN GO TO Q13]

[IF Q12 does not equal "Yes", THEN GO TO Q14]

13. Please select the prescribed **medications** that you took in **the past 24 hours** to <u>treat or manage</u> your current flu/flu-like symptoms? Select all that apply.
    a. Tamiflu (oseltamivir)
    b. Xofluza (baloxavir marboxil)
    c. Relenza (zanamivir)
    d. Oral antibiotics (e.g. Z-pak, amoxicillin, Augmentin, doxycycline)
    e. Inhalers (e.g. albuterol, Ventolin, ProAir, Xopenex, levalbuterol)
    f. Oral steroids (e.g. prednisone, Deltasone, prednisolone, dexamethasone)
    g. Other

14. Did you personally decide to take any **over-the-counter (non-prescription)** medications to <u>manage</u> your current flu or flu-like symptoms in the **past 24 hours**?
    a. Yes
    b. No
    c. I don't know / I can't remember

[IF Q14="Yes", ASK Q15]

15. Which of the following **over-the-counter (non-prescription)** medications did you personally decide to take to <u>treat or manage</u> your current flu or flu-like symptoms in the **past 24 hours**? Select all that apply.

    a. Fever reducers (e.g. ibuprofen, aspirin, Advil, Tylenol, Aleve, acetaminophen)

b. Cough suppressants (e.g. Delsym, Robitussin, dextromethorphan)
c. Chest or mucus decongestants (e.g. Mucinex, guaifenesin)
d. Nasal decongestants (e.g. Sudafed, Sudafed PE, Afrin, Flonase, phenylephrine, pseudoephedrine, fluticasone propionate)
e. I don't know / can't remember
f. Other

Quality of life

16. Please indicate the level of **DIFFICULTY** you had with the following activities in the **past 24 hours**.

	No difficulty at all (0)	A little difficulty (1)	A lot of difficulty (2)
Mobility/getting around			
Self-care activities			
Usual daily activities			

17. Please indicate whether you **EXPERIENCED** the following issues in the **past 24 hours**.

	No experience (0)	Mild experience (1)	Moderate experience (2)	Extreme experience (3)
Pain or discomfort				
Anxiety or depression				

Appendix 2.3 Daily Survey B [participant responded "no" to daily questionnaire]
Please answer the following questions based on your experiences in the past **24 hours**.

Please indicate the level of **DIFFICULTY** you had with the following activities in the **past 24 hours**.

	No difficulty at all (0)	A little difficulty (1)	A lot of difficulty (2)
Mobility/getting around			
Self-care activities			
Usual daily activities			

1. Please indicate whether you **EXPERIENCED** the following issues in the **past 24 hours**.

	No experience (0)	Mild experience (1)	Moderate experience (2)	Extreme experience (3)
Pain or discomfort				
Anxiety or depression				

2. Did you recover from the flu or a flu-like illness in the **past 48 hours**?
   a. No, I haven't had the flu this season
   b. No, I recovered from the flu more than 48 hours ago
   c. Yes

[IF Q3 = "Yes", ASK Q4, otherwise → END SURVEY]

3. Sometimes symptoms can continue even after recovering from a flu illness. Are you **still experiencing** any of the following symptoms?

If you are currently not experiencing a symptom, please select “none”. If you are currently experiencing a symptom, please select mild, moderate, or severe based on how your are currently feeling.

	None (0)	Mild (1)	Moderate (2)	Severe (3)
Cough				
Body/Muscle Ache				
Feeling feverish				
Chills or shivering				
Sweats				
Headache				
Sore throat or itchy/scratchy throat				
Feeling more tired than usual				
Nasal congestion or runny nose				
Sneezing				

Appendix 2.4 – Recovery Survey and flu@home user feedback

Thank you for completing your at-home flu test kit. Please answer the following questions about your flu recovery and your experience using the flu test kit.

1. Do you feel like you have **fully recovered** from the flu or a flu-like illness that you were experiencing?

    a. Yes
    b. No

2. When did your flu or flu-like symptom(s) first start?
    a. [DATE PICKER]

[IF Q1 = "No", SKIP Q3]

3. Approximately when did you feel **fully recovered** from your flu or flu-like illness?
    a. [DATE PICKER]

4. Did you seek medical attention for this **flu or flu-like illness**?
    a. Yes
    b. No

[IF Q4="YES", THEN GO TO Q5]

[IF Q4="NO", THEN GO TO Q14]

5. Where did you seek care from a **healthcare provider**?
    a. Primary care clinic (e.g. family medicine, internal medicine)
    b. Urgent care facility
    c. Ear, nose, and throat (otolaryngology) clinic
    d. Infectious disease clinic
    e. Telemedicine/virtual or online health care services
    f. Phone (*I called my physician or another medical provider*)
    g. Other

6. Did the **healthcare provider** diagnose you as having the flu?
    a. Yes
    b. No
    c. I don't know / I can't remember

[IF Q6="Yes", THEN GO TO Q7]

[IF Q6="No" or "I don't know", THEN GO TO Q8]

7. Did the **healthcare provider** perform any of the following tests to confirm the flu (influenza) diagnosis? Select all that apply.
    a. Nasal swab
    b. Throat swab
    c. Symptoms and examining me only (no test)
    d. I don't know / I can't remember
    e. Other

8. Did the **healthcare provider** <u>diagnose</u> you with a respiratory infection other than the flu (e.g. like the common cold or bronchitis)?
    a. Yes
    b. No

[IF Q8="Yes", ASK Q9 & Q10]

9. What **respiratory infection** were you <u>diagnosed</u> with?
    a. Common cold
    b. Bronchitis
    c. Sore throat (Pharyngitis)
    d. I am not sure
    e. Other: (please specify)

10. Did the **healthcare provider** perform any of the following tests to confirm the respiratory viral infection? Select all that apply.
    a. Nasal swab
    b. Blood test
    c. Throat swab
    d. No test
    e. I don't know / I can't remember
    f. Other: (please specify)

11. Did you go to the <u>emergency room (ER)</u> and/or were you <u>admitted into the hospital</u> for this **flu or flu-like illness**?
    a. Yes, I went to the ER and was then hospitalized
    b. Yes, I only went to the ER
    c. Yes, I was only hospitalized
    d. No, I did not go to the ER nor was I hospitalized

[IF Q11 = "a", ask Q12 & Q13]

[IF Q11 = "b", ONLY ask Q12]

[IF Q11 = "c", ONLY ask Q13]

[IF Q11 = "d", SKIP Q12 & Q13]

12. Approximately when did you <u>visit the ER</u> for your **flu or flu-like illness**?
    a. [DATE PICKER]

13. Approximately when were <u>admitted to the hospital</u> for your **flu or flu-like illness**?
    a. [DATE PICKER]

Medication Usage

This section will ask you about medications that you took during your most recent flu or flu-like illness.

14. Were you prescribed any **medications** to treat or manage your flu or flu-like symptoms?
    a. Yes
    b. No
    c. I don't know / I can't remember

[IF Q14="Yes", THEN GO TO Q15]

[IF Q14 does not equal "Yes", THEN GO TO Q18]

15. Which of the following **medications** were you prescribed by a medical professional to treat or manage your flu or flu-like symptoms?

	I was **NOT** prescribed this medication	I was prescribed this medication and have taken at least 1 dose	I was prescribed this medication and did not take it
Tamiflu (oseltamivir)			
Xofluza (baloxavir marboxil)			
Relenza (zanamivir)			
Peramivir (Rapivab)			
Oral antibiotics (e.g. Z-pak, amoxicillin, Augmentin, doxycycline)			

Inhalers (e.g. albuterol, Ventolin, ProAir, Xopenex, levalbuterol)			
Oral steroids (e.g. prednisone, Deltasone, prednisolone, dexamethasone)			
Yes, but I don't know which medication			
Other			

16. When did you take your first dose of [MEDICATION NAME PULL DOWN FROM Q15; ONE QUESTION PER MEDICATION SELECTED ("I was not prescribed that Medication" was not selected)]
    a. [DATE PICKER]

17. Did you <u>miss at least one dose</u> of [MEDICATION NAME PULL DOWN FROM Q15; ONE QUESTION PER MEDICATION SELECTED("I was not prescribed that medication" was not selected)] that was prescribed to treat or manage your flu illness?
    a. Yes, I did miss at least one dose
    b. No, I did not miss any doses of my prescribed medication

18. Did you take any **over-the-counter (non-prescription)** medications to <u>treat or manage</u> your flu or flu-like symptoms?
    a. Yes
    b. No
    c. I don't know / I can't remember

[IF Q18="Yes", ASK Q19]

[IF Q17 = "No" or "Don't know", SKIP to Q20]

19. Which of the following **over-the-counter (non-prescription)** medications did you take to <u>treat or manage</u> your flu or flu-like symptoms? Select all that apply.

    a. Fever reducers (e.g. ibuprofen, aspirin, Advil, Tylenol, Aleve, acetaminophen)
    b. Cough suppressants (e.g. Delsym, Robitussin, dextromethorphan)

c. Chest or mucus decongestants (e.g. Mucinex, guaifenesin)
d. Nasal decongestants (e.g. Sudafed, Sudafed PE, Afrin, Flonase, phenylephrine, pseudoephedrine, fluticasone propionate)
e. I don’t know / can’t remember
f. Other: (please specify)

Time Missed from Work

20. During your **flu or flu-like illness**, how many hours did you miss from work, school, or your usual daily responsibilities?
    a. [DROP DOWN NUMBERS - 0 - 100]

At-Home Flu Kit

This next section asks questions about your experience with the flu@home test kit and application.

21. The purpose of using flu@home was to:

	Yes (1)	No (0)

Test for the flu		
Give me information about flu and medicine for the flu		
Test different flu medicines		
Participate in a research study about the flu		

22. The instructions in the flu@home app were clear and helpful.
    a. 5 - Strongly agree
    b. 4 - Somewhat agree
    c. 3 - Neither agree nor disagree
    d. 2 - Somewhat disagree
    e. 1 - Strongly disagree

23. Using the flu@home **app** was:
    a. 5 - Very easy
    b. 4 - Easy
    c. 3 - Neutral
    d. 2 - Somewhat difficult
    e. 1 - Very difficult

24. Doing the two flu@home **nasal swab tests** was:
    a. 5 - Very easy
    b. 4 - Easy
    c. 3 - Neutral
    d. 2 - Somewhat difficult
    e. 1 - Very difficult

25. I would have liked it if the flu@home test told me whether or not I had the flu.
    a. 5 - Strongly agree
    b. 4 - Somewhat agree
    c. 3 - Neither agree nor disagree
    d. 2 - Somewhat disagree
    e. 1 - Strongly disagree

26. If the flu@home app were to indicate that you had the flu, which of the following would you have considered doing as next steps? Select all that apply.

	Yes (1)	No (0)
A virtual consultation with a provider (telemedicine visit)		
Share my results anonymously with a national flu tracking system		
Read tips on how to prevent spreading the flu		
Encourage others in my household to use flu@home when ill		

27. I believe it saves time to do a home-based test like flu@home before visiting a healthcare provider.
    a. 5 - Strongly agree
    b. 4 - Somewhat agree
    c. 3 - Neither agree nor disagree
    d. 2 - Somewhat disagree
    e. 1 - Strongly disagree

28. I feel that flu@home test kit could help me better manage my illness.
    a. 5 - Strongly agree
    b. 4 - Somewhat agree
    c. 3 - Neither agree nor disagree
    d. 2 - Somewhat disagree
    e. 1 - Strongly disagree

[END OF SURVEY]

Appendix 3: flu@home app, ©2019 Audere

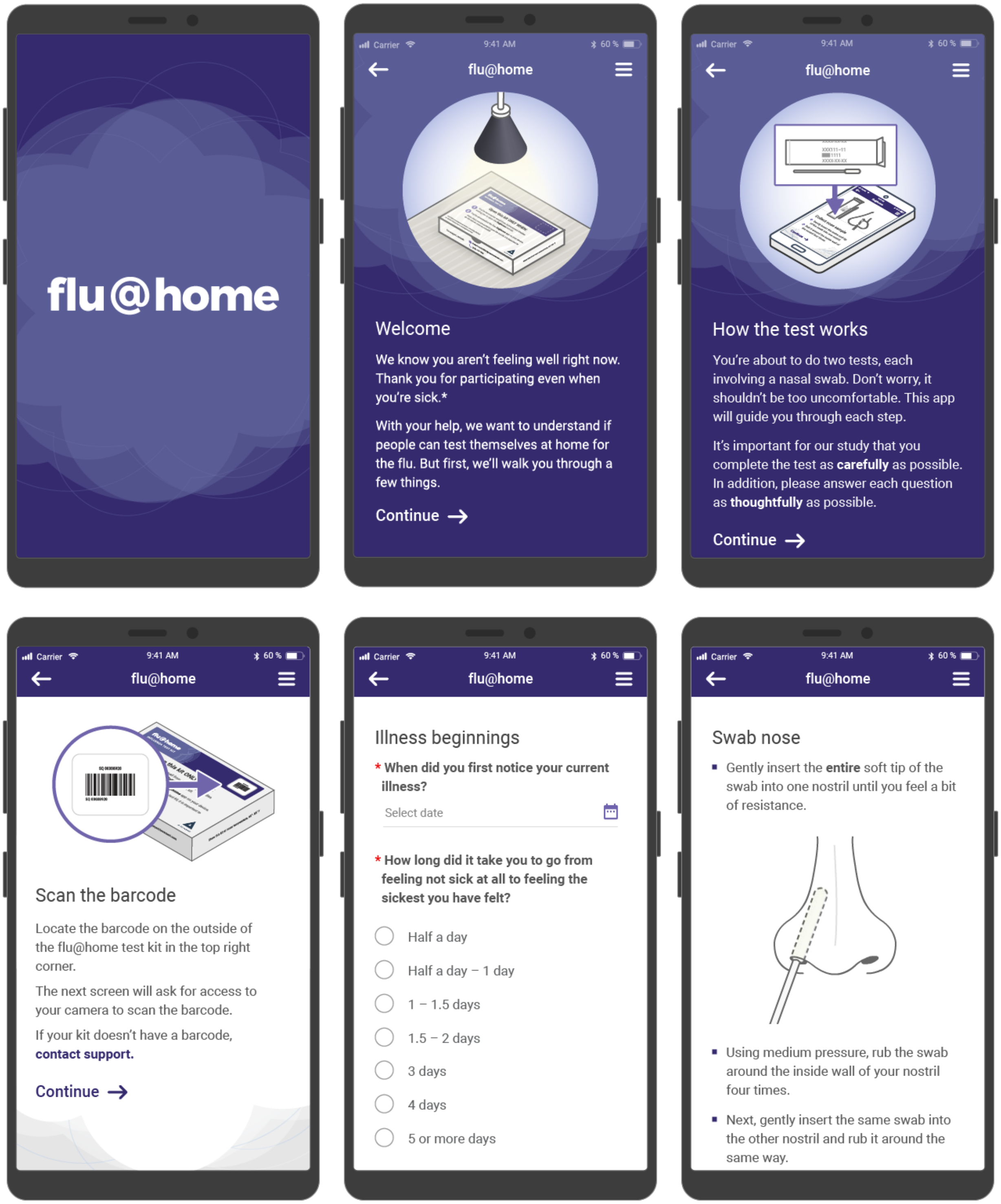

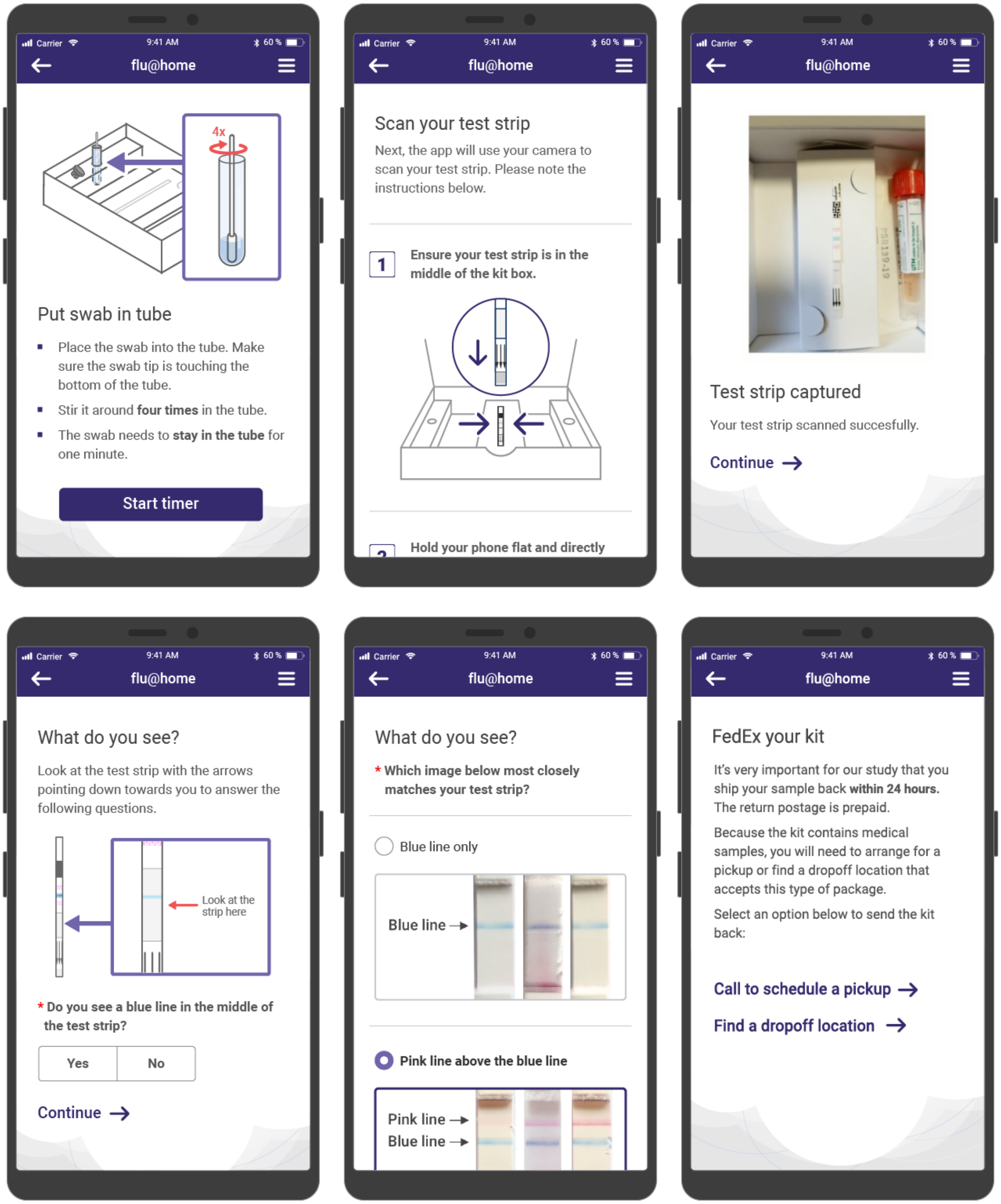
Carrier
9:41 AM
60 %
flu@home
4x
Put swab in tube
Place the swab into the tube. Make sure the swab tip is touching the bottom of the tube.
Stir it around four times in the tube.
The swab needs to stay in the tube for one minute.
Start timer
Carrier
9:41 AM
60 %
flu@home
Scan your test strip
Next, the app will use your camera to scan your test strip. Please note the instructions below.
1
Ensure your test strip is in the middle of the kit box.
2
Hold your phone flat and directly
Carrier
9:41 AM
60 %
flu@home
Test strip captured
Your test strip scanned succesfully.
Continue
Carrier
9:41 AM
60 %
flu@home
What do you see?
Look at the test strip with the arrows pointing down towards you to answer the following questions.
Look at the strip here
* Do you see a blue line in the middle of the test strip?
Yes
No
Continue
Carrier
9:41 AM
60 %
flu@home
What do you see?
* Which image below most closely matches your test strip?
Blue line only
Blue line
Pink line above the blue line
Pink line
Blue line
Carrier
9:41 AM
60 %
flu@home
FedEx your kit
It's very important for our study that you ship your sample back within 24 hours. The return postage is prepaid.
Because the kit contains medical samples, you will need to arrange for a pickup or find a dropoff location that accepts this type of package.
Select an option below to send the kit back:
Call to schedule a pickup
Find a dropoff location

Appendix 4: flu@home in-app illness survey

<u>Appendix 4.1: Symptom Survey</u>

The following questions are very important - your answers will be used to create models for flu prediction. Some questions may look familiar, but we have a goal for each one, so please answer thoughtfully.

1. *Have you experienced any of these new or worsening symptoms in the past **24 hours**? Select all that apply.
    a. Feeling feverish
    b. Headache
    c. Cough
    d. Chills or shivering
    e. Sweats
    f. Sore throat or itchy/scratchy throat
    g. Nausea or vomiting
    h. Runny or stuffy nose
    i. Sneezing
    j. Feeling more tired than usual
    k. Muscle or body aches
    l. Increased trouble with breathing
    m. None of the above

Symptom Severity

2. *How severe were your symptoms? (Select the level of discomfort you felt at the worst point) [Internal note: Ask for every option selected in Q1]
    a. Mild
    b. Moderate
    c. Severe

Illness Beginning

3. *When did you first notice your current illness?
    a. Select date on a calendar: MM/DD/YYYY

4. *How long did it take you to go from feeling not sick at all to feeling the sickest you have felt?
    a. half a day
    b. half a day - 1 day
    c. 1 - 1.5 days
    d. 1.5 - 2 days
    e. 3 days
    f. 4 days
    g. 5 or more days

5. Do you think your current illness is the flu or a common cold?
    a. Flu
    b. Common cold
    c. Another illness

6. Is this illness worse or different from a typical cold for you?
    a. Yes
    b. No

Antiviral Medication

7. *Are you taking an antiviral prescribed by a medical professional for the treatment or prevention of flu?
    a. No
    b. Oseltamivir (Tamiflu)
    c. Zanamivir (Relenza)
    d. Peramivir (Rapivab)
    e. Baloxavir (Xofluza)
    f. Yes, but I don't know which medication
    g. Do not know

[If Q7 = “b-f”, ASK Q8]

8. *When did you start taking the antiviral medication?
    a. [Date picker]

Influenza Vaccination

9. *Have you received this season's influenza (flu) vaccine (since July 1, 2019)? This includes both flu mist nasal spray and the flu shot.
    a. Yes
    b. No
    c. Do not know

[If Q9 = YES, ASK Q10]

10. *When did you get the flu vaccine?
    a. Choose a day, month and year from a calendar control: DD/MM/YYYY

[If Q9 = YES, ASK Q11]

11. *How did you receive the flu vaccine this season (since July 1, 2019)?
    a. Injection (flu shot)
    b. Nasal spray (flu mist)
    c. Do not know

General Health

12. How has your current illness affected your ability to do your regular activities (work, school, etc.)?

a. Not at all
b. A little bit
c. Somewhat
d. Quite a bit
e. Very much

13. Do you currently smoke tobacco?
    a. Yes
    b. No

14. Does anyone in your household currently smoke tobacco?
    a. Yes
    b. No

General Exposure

In this section, the questions are about recent travel and contact with other people.

15. In the past 7 days, have you traveled outside the state of [internal note - *insert state where the kit was sent*]?
    a. Yes
    b. No

16. In the past 7 days, have you visited a country other than the US?
    a. Yes
    b. No, I have not visited a country other than the US

[IF Q16 = YES. ASK Q17]

17. What country or countries did you visit? Select all that apply.
    a. [If YES to response above (Q17),then options to select as many countries as apply, which autocomplete with suggestions as you type the name of the country. Country list: https://seattle-flu-study.slack.com/archives/CL51ARF7G/p1567643084078200]

[If Q16 = NO, ASK Q18]

18. *Where did you spend the majority of your time this past week?
    a. City - text box entry
    b. State - from a list of states
    c. ZIP code - text box entry

19. Including yourself, how many people share your current kitchen or living space?
    a. I live by myself
    b. 2
    c. 3

   d. 4
   e. 5
   f. 6 or more

   [If Q19 = "b-f" (2 or more people), ASK Q20]

20. What age groups of children are in your household? Select all that apply.
    a. No children
    b. Age 0-5
    c. Age 6-12
    d. Age >12

    [If Q20 = "b" (ages 0-5), ASK Q21)

21. Do any children in your household attend daycare or preschool?
    a. Yes
    b. No

    [If Q19 = "b-f" (2 or more people), ASK Q22]

22. In the past 7 days, has someone you live with been diagnosed with the flu by a medical professional?
    a. Yes
    b. No
    c. Don't know

23. Do you believe you were exposed to the flu in the past week?
    a. Yes
    b. No
    c. Don't know

24. In the past week, did you take public transportation?
    a. Yes
    b. No

25. In the past week, were you around sick children?
    a. Yes
    b. No

26. *Are you willing to be contacted for future studies conducted by the same researchers involving materials and steps you complete during this study?
    a. Yes
    b. No

Appendix 4.2: flu@home test and control line presence questions

1. Do you see a blue line in the middle of the test strip?
   a. yes/no
2. Which image below most closely matches your test strip?

a. Blue line only
b. Pink line above the blue line
c. Pink line below the blue line

Appendix 5: Primary state of residence for influenza positive and negative participants

	Overall	Influenza negative	Influenza positive	P
Primary State of Residence	N (%)	N (%)	N (%)	0.229
AK	3 (3)	3 (4)	0 (0.0)	
AL	9 (0.9)	8 (1.0)	1 (0.5)	
AR	6 (0.6)	5 (0.6)	1 (0.5)	
AZ	18 (1.8)	17 (2.2)	1 (0.5)	
CA	70 (7.2)	63 (8.1)	7 (3.5)	
CO	25 (2.6)	20 (2.6)	5 (2.5)	
CT	10 (1.0)	8 (1.0)	2 (1.0)	
DC	4 (0.4)	3 (0.4)	1 (0.5)	
DE	3 (0.3)	2 (0.3)	1 (0.5)	
FL	41 (4.2)	35 (4.5)	6 (3.0)	
GA	23 (2.4)	18 (2.3)	5 (2.5)	
HI	1 (0.1)	1 (0.1)	0 (0.0)	
IA	25 (2.6)	21 (2.7)	4 (2.0)	
ID	11 (1.1)	11 (1.4)	0 (0.0)	
IL	39 (4.0)	26 (3.4)	13 (6.4)	
IN	37 (3.8)	29 (3.7)	8 (4.0)	
KS	15 (1.5)	9 (1.2)	6 (3.0)	
KY	9 (0.9)	8 (1.0)	1 (0.5)	
LA	8 (0.8)	7 (0.9)	1 (0.5)	
MA	22 (2.3)	18 (2.3)	4 (2.0)	
MD	20 (2.0)	15 (1.9)	5 (2.5)	
ME	7 (0.7)	6 (0.8)	1 (0.5)	
MI	56 (5.7)	46 (5.9)	10 (5.0)	
MN	22 (2.3)	15 (1.9)	7 (3.5)	
MO	28 (2.9)	20 (2.6)	8 (4.0)	
MS	6 (0.6)	5 (0.6)	1 (0.5)	
MT	7 (0.7)	5 (0.6)	2 (1.0)	
NC	25 (2.6)	20 (2.6)	5 (2.5)	
ND	5 (0.5)	5 (0.6)	0 (0.0)	
NE	13 (1.3)	10 (1.3)	3 (1.5)	
NH	9 (0.9)	8 (1.0)	1 (0.5)	
NJ	16 (1.6)	9 (1.2)	7 (3.5)	
NM	3 (0.3)	3 (0.4)	0 (0.0)	
NV	10 (1.0)	8 (1.0)	2 (1.0)	
NY	41 (4.2)	30 (3.9)	11 (5.4)	
OH	50 (5.1)	37 (4.8)	13 (6.4)	

OK	8 (0.8)	3 (0.4)	5 (2.5)	
OR	8 (0.8)	6 (0.8)	2 (1.0)	
PA	39 (4.0)	31 (4.0)	8 (4.0)	
RI	4 (0.4)	4 (0.5)	0 (0.0)	
SC	9 (0.9)	8 (1.0)	1 (0.5)	
SD	3 (0.3)	1 (0.1)	2 (1.0)	
TN	20 (2.0)	17 (2.2)	3 (1.5)	
TX	51 (5.2)	40 (5.2)	11 (5.4)	
UT	22 (2.3)	20 (2.6)	2 (1.0)	
VA	25 (2.6)	21 (2.7)	4 (2.0)	
VT	4 (0.4)	4 (0.5)	0 (0.0)	
WA	21 (2.2)	17 (2.2)	4 (2.0)	
WI	39 (4.0)	32 (4.1)	7 (3.5)	
WV	7 (0.7)	4 (0.5)	3 (1.5)	
WY	2 (0.2)	1 (0.1)	1 (0.5)	
NA*	17 (1.7)	11 (1.4)	6 (3.0)	

*"Primary state of residence" question was not answered in the flu@home app survey completed at the time the flu@home kit was used

Appendix 6: Participant responses to flu@home feedback questions

Group	Total Count		Question and count of each Likert-type response (N (%))														
	Total Count		Instructions clear*					App easy to use^					Both nasal swabs were easy*				
	N	NA**	Likert-type response (1-5)					Likert-type response (1-5)					Likert-type response (1-5)				
Overall			1	2	3	4	5	1	2	3	4	5	1	2	3	4	5
	968#			6 (0.6%)	5 (0.5%)	161 (17%)	796 (82%)		4 (0.4%)	21 (2%)	194 (20%)	749 (77%)		18 (2%)	37 (4%)	274 (28%)	639 (66%)
Age			1	2	3	4	5	1	2	3	4	5	1	2	3	4	5
18-24	36	1 (3%)				10 (27%)	25 (70%)				10 (27%)	25 (70%)		2 (6%)	2 (6%)	11 (31%)	20 (55%)
24-34	391	1 (<1%)		1 (0.3%)	2 (0.5%)	52 (13%)	335 (86%)			8 (2%)	64 (16%)	318 (81%)		8 (2%)	14 (4%)	108 (28%)	260 (66%)
35-44	355	6 (2%)		4 (1%)	2 (0.5%)	63 (18%)	280 (79%)		3 (<1%)	8 (2%)	76 (21%)	262 (74%)		6 (2%)	15 (4%)	104 (29%)	224 (63%)
45-54	135					25 (19%)	110 (81%)		3 (2%)		32 (23%)	102 (76%)		1 (<1%)	5 (4%)	36 (27%)	93 (69%)
55+	59			1 (2%)	1 (2%)	11 (19%)	46 (78%)			5 (8%)	12 (20%)	42 (71%)		1 (2%)	1 (2%)	15 (25%)	42 (71%)
Education			1	2	3	4	5	1	2	3	4	5	1	2	3	4	5
High School, GED, or less	46				1 (2%))	4 (9%)	41 (89%)				9 (19%)	37 (80%)			2 (4%)	9 (19%)	35 (76%)
Some college	206	4 (2%)		4 (2%)	1 (<1%)	28 (14%)	172 (83%)		1 (<1%)	6 (3%)	31 (15%)	164 (80%)		5 (2%)	5 (2%)	55 (26%)	137 (67%)
Bachelors or equivalent degree	484	2 (<1%)		2 (<1%)	1 (<1%)	91 (19%)	387 (80%)		1 (<1%)	9 (2%)	104 (20%)	368 (76%)		6 (1%)	19 (4%)	144 (30%)	313 65%)
Graduate or masters degree	199	1 (<1%)		1 (<1%)	1 (<1%)	30 (15%)	165 (83%)		2 (1%)	3 (2%)	38 (20%)	155 (77%)		5 (3%)	9 (5%)	53 (27%)	131 (66%)
Doctorate degree	37	1 (3%)		1 (3%)	1 (3%)	7 (19%)	28 (76%)			3 (8%)	12 (32%)	21 (57%)		2 (5%)	2 (5%)	12 (32%)	20 (54%)
No answer	4					1 (25%)	3 (75%)					4 (100%)				1 (25%)	3 (75%)
PCR +/-			1	2	3	4	5	1	2	3	4	5	1	2	3	4	5
Negative	774	7 <1%)		3 (<1%)	3 (<1%)	115 (15%)	646 (84%)		2 (<1%)	17 (2%)	145 (19%)	603 (78%)		12 (2%)	28 (4%)	210 (27%)	517 (67%)
Positive	202	1 (<1%)		3 (2%)	2 (1%)	46 (23%)	150 (74%)		2 (1%)	4 (2%)	49 (24%)	146 (72%)		6 (3%)	9 (5%)	64 (32%)	122 (60%)
User/expert disagree vs. agree			1	2	3	4	5	1	2	3	4	5	1	2	3	4	5
Agree	932	8 (<1%)		6 (<1%)	4 (<1%)	146 (16%)	768 (82%)		4 (<1%)	16 (2%)	182 (20%)	722 (78%)		18 (2%)	33 (4%)	257 (28%)	616 (66%)
Disagree	44				1 (2%)	15 (34%)	28 (64%)			5 (11%)	12 (27%)	27 (61%)			4 (9%)	17 (39%)	23 (52%)

*Likert-type responses: (1) = Strongly Disagree, (2) = Disagree, (3) = Neither agree nor disagree, (4) = Somewhat agree, (5) = Strongly agree; ^Likert-type responses: (1) Very Difficult, (2) Somewhat difficult, (3) Neutral, (4) Easy, (5) Very Easy; ** NA = no answer; #8 participants did not complete the survey.

**Appendix 7: Severity of individual symptoms in participants with and without influenza confirmed by laboratory testing**				
**Symptom & Severity**	**Overall**	**Influenza negative**	**Influenza positive**	**P**
**Fever (total reported)**	**441**	**304**	**137**	<0.001
mild	206 (46.7)	168 (55.3)	38 (27.7)	
moderate	187 (42.4)	122 (40.1)	65 (47.4)	
severe	48 (10.9)	14 (4.6)	34 (24.8)	
**Headache**	**672**	**522**	**150**	0.001
mild	225 (33.5)	183 (35.1)	42 (28.0)	
moderate	334 (49.7)	266 (51.0)	68 (45.3)	
severe	113 (16.8)	73 (14.0)	40 (26.7)	
**Cough**	**789**	**610**	**179**	<0.001
mild	324 (41.1)	289 (47.4)	35 (19.6)	
moderate	369 (46.8)	264 (43.3)	105 (58.7)	
severe	96 (12.2)	57 (9.3)	39 (21.8)	
**Chills/Shivering**	402	270	132	<0.001
mild	169 (42.0)	136 (50.4)	33 (25.0)	
moderate	179 (44.5)	108 (40.0)	71 (53.8)	
severe	54 (13.4)	26 (9.6)	28 (21.2)	
**Sweats**	**352**	**250**	**102**	0.002
mild	155 (44.0)	124 (49.6)	31 (30.4)	
moderate	156 (44.3)	97 (38.8)	59 (57.8)	
severe	41 (11.6)	29 (11.6)	12 (11.8)	
**Sore Throat**	**725**	**569**	**156**	0.007
mild	235 (32.4)	200 (35.1)	35 (22.4)	
moderate	371 (51.2)	283 (49.7)	88 (56.4)	
severe	119 (16.4)	86 (15.1)	33	
**Vomiting**	**153**	**111**	**42**	0.658
mild	82 (53.6)	61 (55.0)	21 (50.0)	
moderate	61 (39.9)	42 (37.8)	19 (45.2)	
severe	10 (6.5)	8 (7.2)	2 (4.8)	
**Runny Nose**	**741**	**581**	**160**	0.104
mild	231 (31.2)	185 (31.8)	46 (28.7)	
moderate	364 (49.1)	291 (50.1)	73 (45.6)	
severe	146 (19.7)	105 (18.1)	41 (25.6)	
**Sneezing**	**577**	**466**	**111**	0.957
mild	316 (54.8)	254 (54.5)	62 (55.9)	
moderate	215 (37.3)	175 (37.6)	40 (36.0)	
severe	46 (8.0)	37 (7.9)	9 (8.1)	
**Fatigue**	**784**	**617**	**167**	<0.001
mild	156 (19.9)	136 (22.0)	20 (12.0)	

moderate	421 (53.7)	339 (54.9)	82 (49.1)	
severe	207 (26.4)	142 (23.0)	65 (38.9)	
**Muscle/Body Aches**	**597**	**447**	**150**	<0.001
mild	168 (28.1)	141 (31.5)	27 (18.0)	
moderate	318 (53.3)	246 (55.0)	72 (48.0)	
severe	111 (18.6)	60 (13.4)	51 (34.0)	
**Trouble Breathing**	**313**	**235**	**78**	0.007
mild	130 (41.5)	109 (46.4)	21 (26.9)	
moderate	154 (49.2)	104 (44.3)	50 (64.1)	
severe	29 (9.3)	22 (9.4)	7 (9.0)	

Appendix 8: Disagreement over index test results between participants and experts and count of each error type.

	Participant index test	Expert index test	PCR result	Verbatim error made by participant or expert	Count
**Participant errors**					
**False positive**					
	+A	(-)	(-)	Participant said flu A, expert said negative, PCR was negative	8
	+B	(-)	(-)	Participant said flu B, expert said negative, PCR was negative	7
**Dual positive**					
	+A & +B	(-)	(-)	Participant said flu A and B, expert said negative, PCR was negative	4
	+A & +B	A+	A+	Participant said flu A and B, expert said positive for flu A, PCR was flu A	1
	+A & +B	Inv	(-)	Participant said flu A and B, expert said invalid, PCR was negative	2
	+A & +B	Inv	+	Participant said flu A and B, expert said invalid, PCR was positive	1
**False negative**					
	(-)	+A	+A	Participant said negative, expert said flu A, PCR was flu A	4
	(-)	+B	+B	Participant said negative, expert said flu B, PCR was flu B	0
**False invalid**					
	Inv	(-)	N/A	Participant said invalid, expert said valid but negative (PCR result not applicable to error determination)	3
**Wrong type of flu**					
	+B	+A	+A	Participant said flu B, expert said flu A, PCR was flu A	0
	+A	+B	+B	Participant said flu A, expert said flu B, PCR was flu B	0
	+A	(-)	+B	Participant said flu A, expert said negative, PCR was flu B	2
	+B	(-)	+A	Participant said flu B expert said negative, PCR was flu A	4
				Total participant errors	**36**
**Expert errors**					
**False negative**					
	+A	(-)	+A	Participant said flu A, expert said negative, PCR was flu A	4
	+B	(-)	+B	Participant said flu B, expert said negative, PCR was flu B	2
**False positive**					
	(-)	+A	(-)	Participant said negative, expert said flu A, PCR was negative	2
				**Total expert errors**	**8**
				**Total participant and expert disagreements**	**44**

"A" = influenza A; "B" = influenza B; "Inv" = Invalid; "(-)" = negative; "+" = positive
